# Machine learning-based identification of high-risk bone metastasis factors after radical prostatectomy in prostate cancer

**DOI:** 10.3389/fonc.2025.1549851

**Published:** 2025-02-19

**Authors:** Haijun Yang, Chengxiang Wei, Shan Zhou, Fei Mao

**Affiliations:** ^1^ Department of Urology, Huai’an Hongze District People’s Hospital, Huai’an, Jiangsu, China; ^2^ Department of Ultrasound, The Affiliated Huaian No. 1 People’s Hospital of Nanjing Medical University, Huai’an, China; ^3^ Department of Urology, Huaian Clinical College of Xuzhou Medical University, Huai’an, China; ^4^ Department of Urology, The Affiliated Huai’an No.1 People’s Hospital of Nanjing Medical University, Huai’an, China

**Keywords:** prostate cancer, bone metastasis, prognosis, risk factor, machine learning

## Abstract

**Background:**

Bone metastasis is a serious complication following radical prostatectomy in prostate cancer patients, significantly affecting their long-term survival. This study aims to develop a clinical predictive model utilizing Magnetic Resonance Imaging (MRI) and advanced machine learning algorithms to identify key factors that increase the risk of bone metastasis (BM).

**Patients and methods:**

The study analyzed a cohort of 1161 prostate cancer patients, including 38 who developed bone metastasis. Preoperative T2-weighted images (T2WI) were obtained, and tumor lesions were manually delineated to extract relevant features from the imaging data. Spearman correlation analysis, the least absolute shrinkage and selection operator (LASSO) algorithm, and logistic regression were used to select and construct the model. Four machine learning algorithms—extreme gradient boosting (XGBoost), random forest (RF), support vector machine (SVM), and k-nearest neighbor (KNN)—were employed to predict BM occurrence, integrating these with clinical information.

**Results:**

Among the four prognostic models evaluated, the XGBoost algorithm performed the best. In the training dataset, the XGBoost model achieved an AUC of 0.926 (0.870-0.982), an accuracy of 0.847 (0.773-0.921), a sensitivity of 0.880 (0.835-0.926), and a specificity of 0.829 (0.755-0.904). In the validation dataset, the XGBoost model attained an AUC of 0.706 (0.586-0.826), an accuracy of 0.687 (0.661-0.713), a sensitivity of 0.693 (0.557-0.829), and a specificity of 0.664 (0.505-0.822). The external validation dataset yielded an AUC of 0.91, demonstrating the robust predictive capabilities of the XGBoost model.

**Conclusion:**

The predictive model for bone metastasis in prostate cancer, developed using the XGBoost machine learning algorithm, shows high accuracy and significant clinical relevance. This model provides a valuable tool for identifying high-risk patients, potentially informing better management and treatment strategies.

## Background

Prostate cancer stands as one of the most prevalent malignancies affecting men, presenting a significant challenge in achieving long-term survival (1). With the global population aging, the incidence of prostate cancer is anticipated to rise steadily (2). In areas lacking effective cancer prevention and treatment strategies and with limited healthcare resources, the efficacy of prostate cancer treatment becomes even more critical.

Prostate cancer has a unique tendency to metastasize to bone more frequently than to visceral organs (3). This presents a significant challenge for clinicians, as patients with bone metastasis (BM) face a systemic malignancy often managed only with palliative care. BM can lead to severe complications such as bone pain, pathological fractures, and physical disabilities (4). Additionally, BM may cause systemic issues such as hypercalcemia, leading to neurological, cardiovascular, and other health problems that severely impact quality of life (5).

Recent advancements have improved our understanding of prostate cancer’s pathogenesis and clinical management. Raboy’s pioneering work on radical prostatectomy has established it as an effective treatment, known for its improved surgical safety and oncological outcomes (6). Minimally invasive techniques have further reduced postoperative complications. Moreover, new medical technologies have introduced various anti-cancer treatments, progressively reducing BM incidence and enhancing long-term survival rates.

Despite these advancements, some patients still experience metastasis, with BM being particularly difficult to treat. Early detection and intervention are crucial in managing BM from prostate cancer. Diagnostic methods such as single photon emission computed tomography (SPECT) and positron emission tomography-computed tomography (PET-CT) are widely used for diagnosing BM (7). While these methods are highly effective in identifying BM lesions and their effects on surrounding tissues, they also contribute to significant financial burdens for patients and healthcare systems.

Artificial intelligence (AI) holds significant promise in medicine, especially through machine learning algorithms. These algorithms can analyze large datasets to uncover complex patterns and relationships, offering enhanced predictive capabilities for disease outcomes (8, 9). Unlike traditional predictive methods based on statistical models and heuristic rules, machine learning algorithms are adaptable and can handle diverse data complexities, minimizing errors due to researcher subjectivity and methodological constraints.

In this study, we employ an AI framework to analyze clinical profiles and imaging datasets of prostate cancer patients, aiming to predict high-risk factors for postoperative BM. This dual approach not only aids clinicians in identifying at-risk patients more swiftly but also plays a crucial role in developing precise and personalized diagnostic and therapeutic strategies.

## Materials and methods

### Study subjects

This study utilized clinical imaging data from Huai’an Hongze District People’s Hospital and Huai’an First People’s Hospital.

Inclusion Criteria:

1. Patients who underwent robotic-assisted radical prostatectomy or laparoscopic-assisted radical prostatectomy.
2. The surgical team included experienced surgeons skilled in performing in-house radical prostatectomy procedures.
3. Patients diagnosed with prostate cancer bone metastasis (BM) through surgical exploration, ECT/PET-CT imaging, or pathological biopsy.
4. Patients who had undergone preoperative prostate MRI.

Exclusion Criteria:

1. Patients with concomitant diagnoses of other malignant tumors.
2. Patients with other distant metastases, such as prostate cancer lung metastases.
3. Patients with severe organic disorders affecting the liver or kidneys.
4. Patients with a history of autoimmune diseases.
5. Patients with a history of steroid medication use.
6. Patients with documented viral or bacterial infections during preoperative evaluations.
7. Patients with incomplete case documentation, missing clinical details, non-evaluable images due to poor quality, or those who were lost to follow-up.

All patients included in this study were followed up postoperatively until June 2023.

### Data preprocessing

Prostate cancer patients treated between January 2015 and January 2021 in Huai’an Hongze District People’s Hospital were selected as the internal validation cohort. Simultaneously, prostate cancer patients from Huai’an First People’s Hospital during the same period were designated as the external validation cohort. For the internal validation cohort, the data were randomly divided into a training subset (70%) and a test subset (30%).

### Clinical imaging methods

A 3.0T MRI scanner (MAGNETOM Verio, Siemens Healthcare, Erlangen, Germany) was utilized for this study. The imaging process was overseen by a skilled imaging physician, who adhered to a standardized technical protocol. Prior to the scan, the physician instructed the patient to drink a moderate amount of water to ensure the bladder was sufficiently filled without causing an urgent need to urinate. The patient was positioned supine with a head-first orientation, and scanning of the pelvis was performed using an abdominal phased-array coil. T2-weighted images (T2WI) obtained from multiparametric MRI (mpMRI) were used to construct the predictive model for bone metastasis (BM) in prostate cancer.

### Imaging histology modeling

Two radiologists, each with over five years of experience in diagnosing prostate cancer, carefully imported the multiparametric MRI (mpMRI) images into MRIcroGL software. They meticulously delineated three-dimensional regions of interest (VOIs) around the tumor, ensuring that the contours did not extend beyond the tumor boundaries. In cases where significant discrepancies were noted between the VOIs marked by the two radiologists, an additional imaging specialist was brought in to revise the VOIs. Following a thorough discussion, a consensus was achieved to finalize the definitive VOIs.

This study adhered to the guidelines established by the Image Biomarker Standardization Initiative (IBSI) and employed the PyRadiomics v3.0.1 package within Python 3.6 for extracting imageomics features from the images in the internal validation cohort. A crucial step involved normalizing and standardizing the various imageomics features. This process aimed to harmonize the scales, units, and ranges across different image datasets, improving their comparability and facilitating integration into analytical models. Additionally, the data were remapped into specific ranges to optimize them for further processing and training. Spearman correlation analysis was used to exclude features with a correlation coefficient of ≥ 0.70. The remaining features were then refined using the least absolute shrinkage and selection operator (LASSO) with 10-fold cross-validation.

An imaging histology model will be developed through logistic regression analysis, utilizing the meticulously selected imaging histology feature data. The imaging histology score (Radscore) for each patient will be computed, and graphical representations of the Radscore distributions for both the training and test datasets will be generated.

### Clinical data collection and screening of clinical variables

A comprehensive dataset of 33 variables was compiled, encompassing preoperative, intraoperative, and postoperative factors.

Preoperative Variables:

- Patient Demographics: Age, smoking history, alcohol abuse history, and body mass index (BMI).
- Clinical Attributes: American Society of Anesthesiologists (ASA) score, Nutritional Risk Screening 2002 (NRS2002) score, history of previous surgeries, family medical history, and history of endocrine therapy.
- Medical History: Conditions such as anemia, diabetes mellitus, hypertension, hyperlipidemia, and coronary heart disease (CHD).
- Tumor-Specific Characteristics: Pathological Gleason score, T-stage, N-stage, extracapsular extension (ECE), peripheral nerve infiltration (PNI), site of cancer invasion, pathological tumor volume, and laboratory markers including albumin (ALB), Prostate-Specific Antigen (PSA), and Alkaline Phosphatase (ALP).

Intraoperative Variables:

- Surgical approach, duration of surgery, intraoperative blood loss, and whether lymph node dissection was performed.

Postoperative Variables:

- Laboratory indicators measured within 48 hours post-operation included PSA, procalcitonin (PCT), C-Reactive Protein (CRP), neutrophil-to-lymphocyte ratio (NLR), and serum amyloid A (SAA). PSA levels were measured 6 months after the operation.

The primary outcome variable of interest was the development of prostate cancer bone metastasis (BM). To identify independent predictors of BM, univariate analysis was performed on the internal validation set, followed by logistic regression analysis of relevant variables.

Subsequently, four distinct machine learning models—Extreme Gradient Boosting (XGBoost), Random Forest (RF), Support Vector Machine (SVM), and K-Nearest Neighbors (KNN)—were employed to evaluate the significance of each clinical feature and rank them based on their weighted importance. Features that ranked within the top ten across all four models and demonstrated statistical significance in both univariate and multivariate analyses were selected for further evaluation.

### Construction and evaluation of predictive models for machine learning algorithms

The selected clinical variables and Radscore were integrated into four machine learning algorithms—Support Vector Machine (SVM), Random Forest (RF), Extreme Gradient Boosting (XGBoost), and K-Nearest Neighbors (KNN)—for predictive modeling. Receiver Operating Characteristic (ROC) curves were generated to compute the Area Under the Curve (AUC) values, assessing the predictive performance of each model. Calibration curves were plotted to evaluate the models’ practical utility, and Decision Curve Analysis (DCA) was performed to measure the clinical benefits of the models in guiding interventional therapy.

For internal validation, k-fold cross-validation was used to ensure robust performance assessment. The model that demonstrated the best performance in internal validation was then subjected to external validation using an independent test dataset. ROC curves were again constructed to evaluate the model’s generalizability and predictive accuracy on the external dataset.

To enhance the interpretability of the model, Shapley Additive Explanations (SHAP) summary plots were employed to rank the importance of risk factors. Additionally, single-sample SHAP plots were used to analyze and understand the prediction outcomes for individual samples.

## Results

### Basic clinical information of the patient

**Figure 1:**
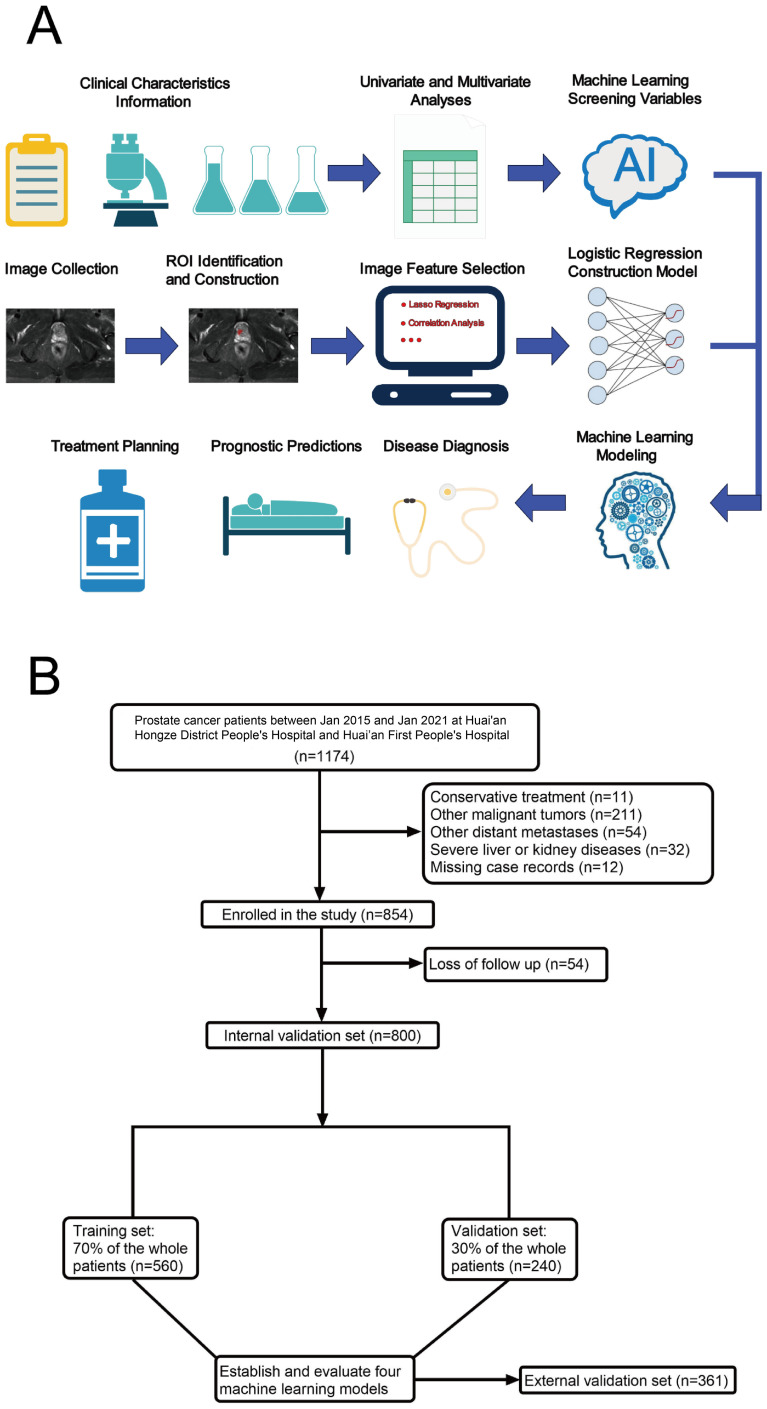
Model-making process and flowchart of the study. **(A)** Study design flow chart. **(B)** Flow diagram of patients included in the study.

The study involved 1,161 prostate cancer patients, of whom 38 (3.27%) had prostate cancer bone metastasis (BM) (refer to **Figures 1A, B**). Within this cohort, 800 patients were allocated to the internal validation set, with 25 cases (3.125%) identified as having prostate cancer BM. The external validation set included 361 patients, among whom 13 (3.60%) had prostate cancer BM. Detailed data from the study are presented in **Supplementary Table S1**.

### Imaging histology feature screening and modeling

**Figure 2:**
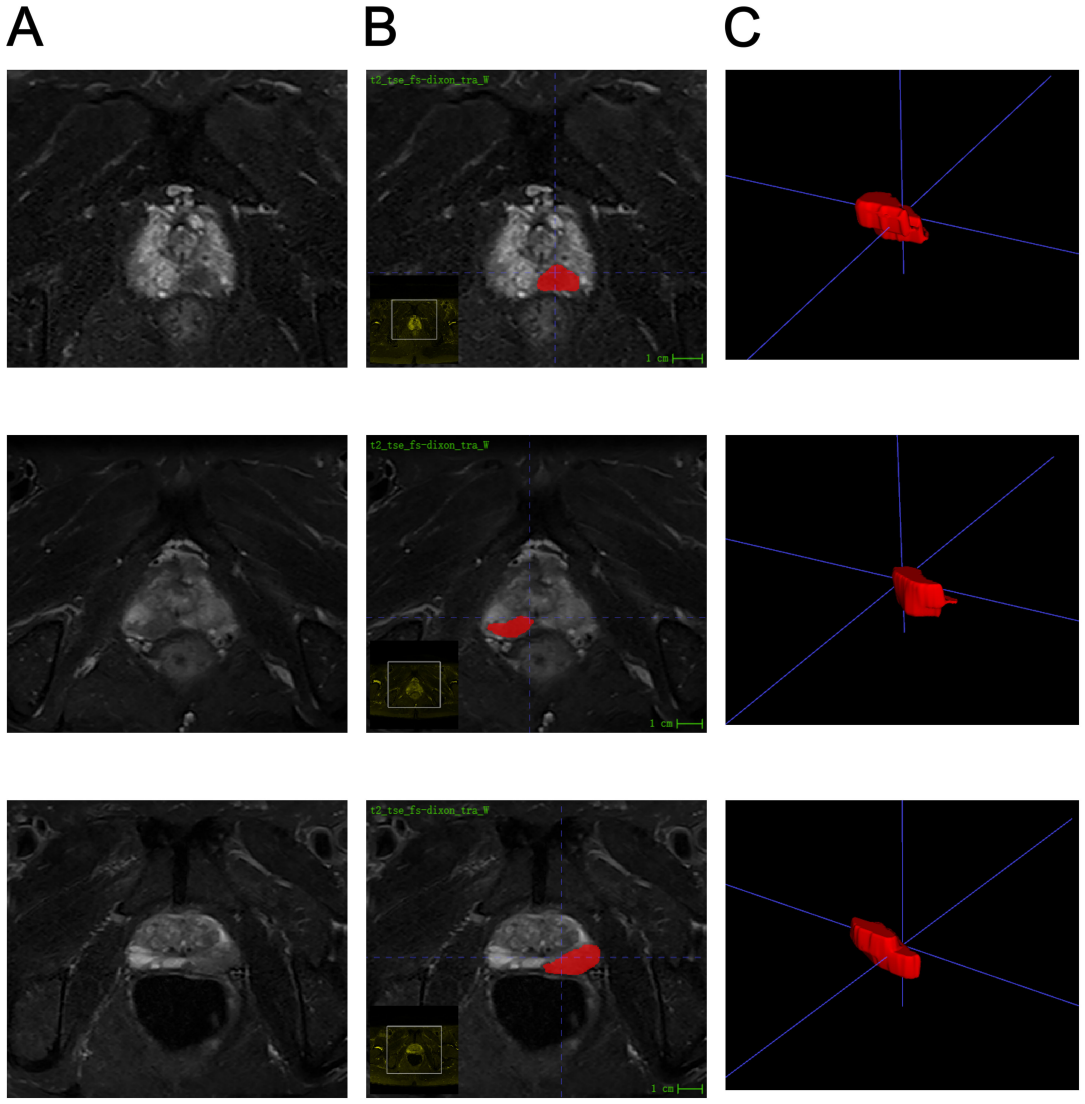
Schematic representation of ROI profiles. **(A)** Illustrates the T2WI sequence featuring prostate cancer situated in the peripheral zone. **(B)** Describes the ROI profile of a patient with bone metastasis from prostate cancer. **(C)** Shows the 3D ROI contour of the tumor foci of a patient with bone metastasis from prostate cancer.

**Figure 3:**
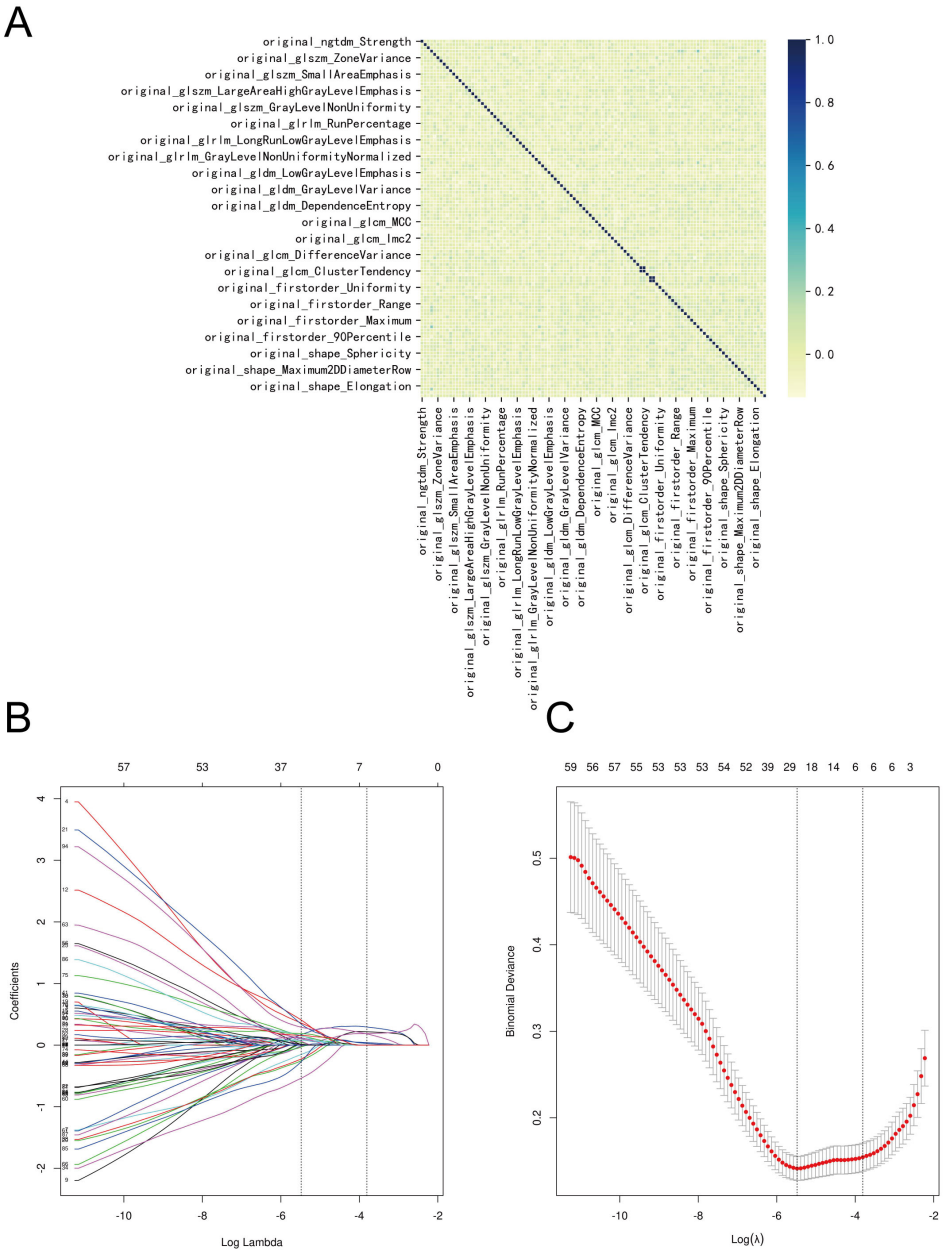
Screening process for imaging histologic features. **(A)** Correlation of imaging histologic features. **(B)** The Lasso coefficient profile of 100 features, with each curve representing a feature. **(C)** The most optimal parameter selected in Lasso regression by using the 10-fold cross-validation. Red dots indicate the likelihood of deviance values, gray lines represent the standard error (SE), and vertical dot lines correspond to optimal values by minimum criteria, and 1-SE, respectively.

Two radiologists meticulously delineated the three-dimensional tumor regions layer by layer (see **Figures 2A–C**). Spearman correlation analysis was employed to investigate the relationships among imaging histological variables, resulting in the exclusion of nine variables with significant correlations (**Figure 3A**). The remaining features were further refined using the Lasso regression algorithm with 10-fold cross-validation. A λ value of 0.022 was applied, leading to the removal of 94 features. The final set of significant features included original_glszm_LargeAreaHighGrayLevelEmphasis, original_glrlm_ShortRunHighGrayLevelEmphasis, original_gldm_SmallDependenceHighGrayLevelEmphasis, original_glcm_SumAverage, original_glcm_DifferenceEntropy, and original_shape_Maximum2DDiameterColumn (see **Figures 3B, C**).

**Figure 4:**
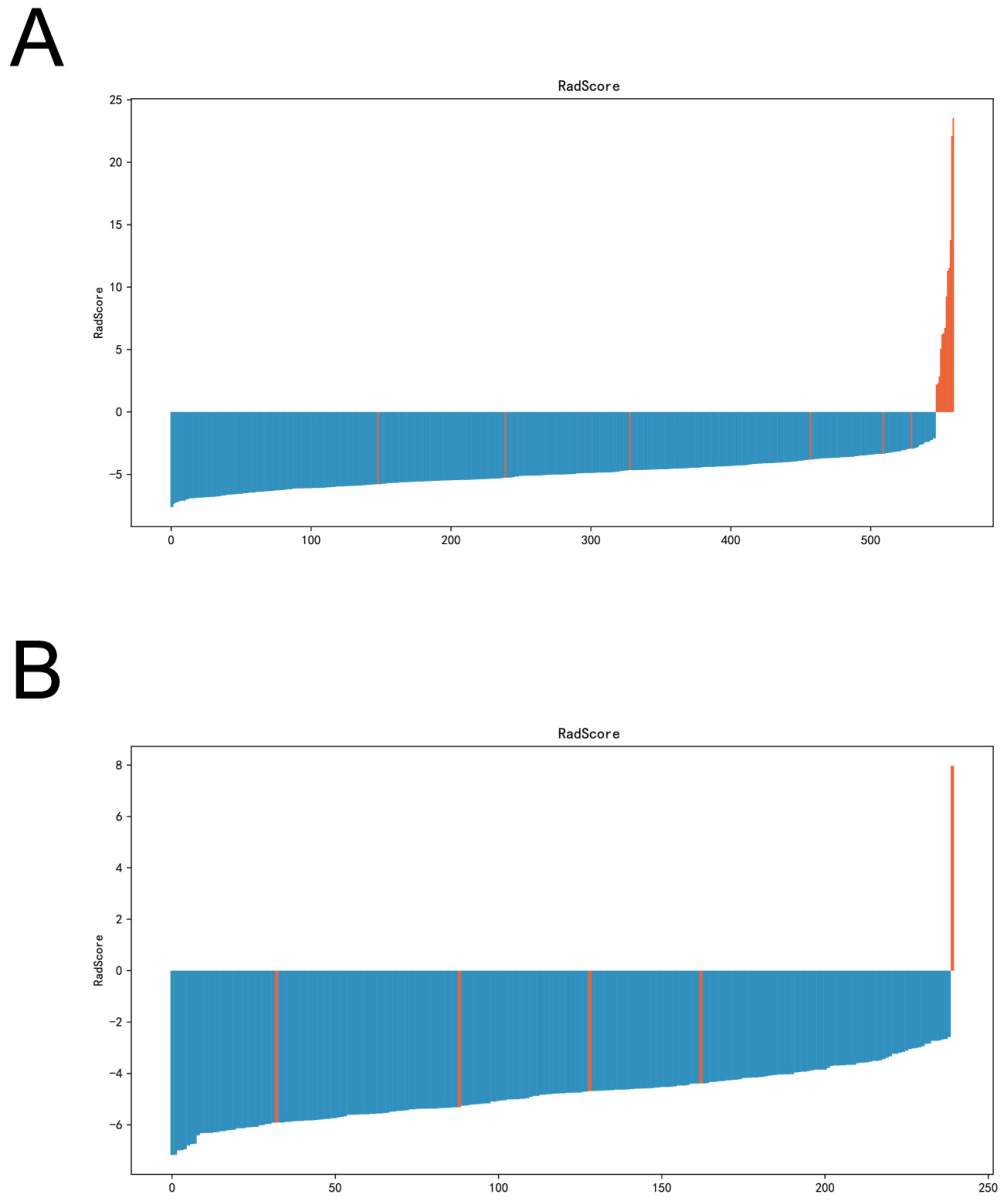
Radscore plots of the imagingomics model in the training and validation sets. **(A)** Radscore plots of imaging histology models in the training set. **(B)** Imaging histology model radscore plots in the validation set.

An imaging histology model was constructed using these six key features. The Radscore for each patient was computed using the following formula: Radscore= -0.8829567743441374 * original_shape_Maximum2DDiameterColumn - 0.07422776022174774 * original_glcm_DifferenceEntropy + 0.03633822348870328 * original_glcm_SumAverage + 1.0492049674143458 * original_gldm_ SmallDependenceHighGrayLevelEmphasis + 0.07224865665312416 * original_glrlm_ShortRunHighGrayLevelEmphasis + 0.00011730863502181296 * original_glszm_LargeAreaaHighGrayLevelEmphasis - 0.12357927365537154 (**Figures 4A, B**).

### Screening of clinical risk factors for bone metastases from prostate cancer

Both univariate and multivariate analyses revealed that several factors—specifically, ALP levels, Gleason Score, extracapsular extension (ECE) invasion, pathological tumor volume, preoperative PSA levels, postoperative PSA levels, and postoperative procalcitonin (PCT) values—significantly influenced the occurrence of postoperative bone metastasis (BM) in prostate cancer, with all factors showing statistical significance (P<0.05) (see **Table 1**).

**Table 1:** Univariate and multivariate analysis of variables related to BM.

Variables		Univariate analysis			Multivariate analysis		
		OR	95%CI	P-value	OR	95%CI	P-value
Age	<65	Reference					
	≥65	1.342	[0.572, 3.148]	0.499			
BMI	<25 kg/m^2^	Reference					
	≥25 kg/m^2^	1.21	[0.515, 2.845]	0.662			
ASA	<3	Reference					
	≥3	1.547	[0.693, 3.456]	0.287			
ALB	≥30 g/l	Reference					
	<30 g/l	1.578	[0.711, 3.506]	0.262			
NRS2002 score	<3	Reference			Reference		
	≥3	2.622	[1.163, 5.915]	0.02	1.69	[0.571, 5.082]	0.339
ALP	<360 U/L	Reference			Reference		
	≥360 U/L	3.493	[1.564, 7.801]	0.002	4.441	[1.494, 13.419]	0.007
Family history	No	Reference			Reference		
	Yes	2.791	[1.207, 6.452]	0.016	2.106	[0.619, 6.747]	0.215
Drinking history	No	Reference			Reference		
	Yes	2.305	[1.030, 5.162]	0.042	2.18	[0.74, 6.354]	0.15
Smoking history	No	Reference					
	Yes	2.16	[0.971, 4.806]	0.059			
Prostate electrosurgery history	No	Reference					
	Yes	1.285	[0.529, 3.127]	0.58			
Anemia	No	Reference					
	Yes	2.197	[0.953, 5.063]	0.065			
Hyperlipidemia	No	Reference					
	Yes	1.016	[0.375, 2.751]	0.975			
Hypertension	No	Reference			Reference		
	Yes	2.655	[1.184, 5.951]	0.018	2.154	[0.664, 6.624]	0.185
Diabetes	No	Reference					
	Yes	2.232	[0.968, 5.146]	0.06			
CHD	No	Reference			Reference		
	Yes	3.696	[1.423, 9.599]	0.007	2.552	[0.553, 10.148]	0.2
Gleason Score	<8	Reference			Reference		
	≥8	5.182	[2.314, 11.607]	<0.001	6.184	[1.952, 20.572]	0.002
ECE	No	Reference			Reference		
	Yes	5.521	[2.204, 13.826]	<0.001	5.576	[1.297, 22.667]	0.017
Endocrine therapy	No	Reference					
	Yes	1.64	[0.726, 3.705]	0.234			
Cancer tissue invasion site	Non-tip and bottom	Reference			Reference		
	Tip and bottom	2.701	[1.139, 6.404]	0.024	2.365	[0.575, 8.899]	0.211
Pathological tumour volume	<6cc	Reference			Reference		
	≥6cc	3.548	[1.588, 7.925]	0.002	8.629	[2.687, 30.993]	<0.001
T-stage	T1~T2	Reference					
	T3~T4	2.01	[0.851, 4.748]	0.111			
N-stage	N0	Reference					
	N1	1.687	[0.619, 4.597]	0.306			
PNI	No	Reference			Reference		
	Yes	2.815	[1.186, 6.679]	0.019	2.027	[0.584, 6.424]	0.242
Preoperative PSA	<20 ng/ml	Reference			Reference		
	≥20 ng/ml	3.988	[1.748, 9.100]	0.001	6.11	[1.842, 21.048]	0.003
Postoperative PSA	<0.2 ng/ml	Reference			Reference		
	≥0.2 ng/ml	5.184	[2.291, 11.732]	<0.001	4.011	[1.238, 12.712]	0.018
Surgical procedure	Laparoscopic surgery	Reference					
	Robotic surgery	1.064	[0.479, 2.361]	0.879			
Lymph node dissection	No	Reference					
	Yes	0.882	[0.391, 1.990]	0.763			
Surgery time	<270 min	Reference			Reference		
	≥270 min	2.436	[1.094, 5.422]	0.029	2.476	[0.822, 7.48]	0.103
Intraoperative bleeding	<100 ml	Reference					
	≥100 ml	1.889	[0.846, 4.214]	0.12			
PCT level	<0.05 ng/ml	Reference			Reference		
	≥0.05 ng/ml	5.174	[2.303, 11.622]	<0.001	4.84	[1.622, 15.012]	0.005
CRP level	<10 mg/l	Reference			Reference		
	≥10 mg/l	2.529	[1.095, 5.840]	0.03	2.493	[0.726, 8.146]	0.133
SAA level	<10 mg/l	Reference			Reference		
	≥10 mg/l	2.427	[1.071, 5.501]	0.034	2.921	[0.951, 8.938]	0.057
NLR	<3	Reference					
	≥3	1.867	[0.791, 4.406]	0.154			

OR, odds ratio; CI, confidence interval; BMI, body mass index; ASA, the American Society of Anesthesiologists; ALB, albumin; PCT, procalcitonin; CRP, C-reactive protein; SAA, serum amyloid A; NRS2002, nutrition risk screening 2002; CHD, coronary heart disease; ALP, alkaline phosphatase; NLR, neutrophil-to-lymphocyte ratio; ECE, extracapsular extension; PNI, peripheral nerve invasion; PSA, Prostate-specific antigen.

**Figure 5:**
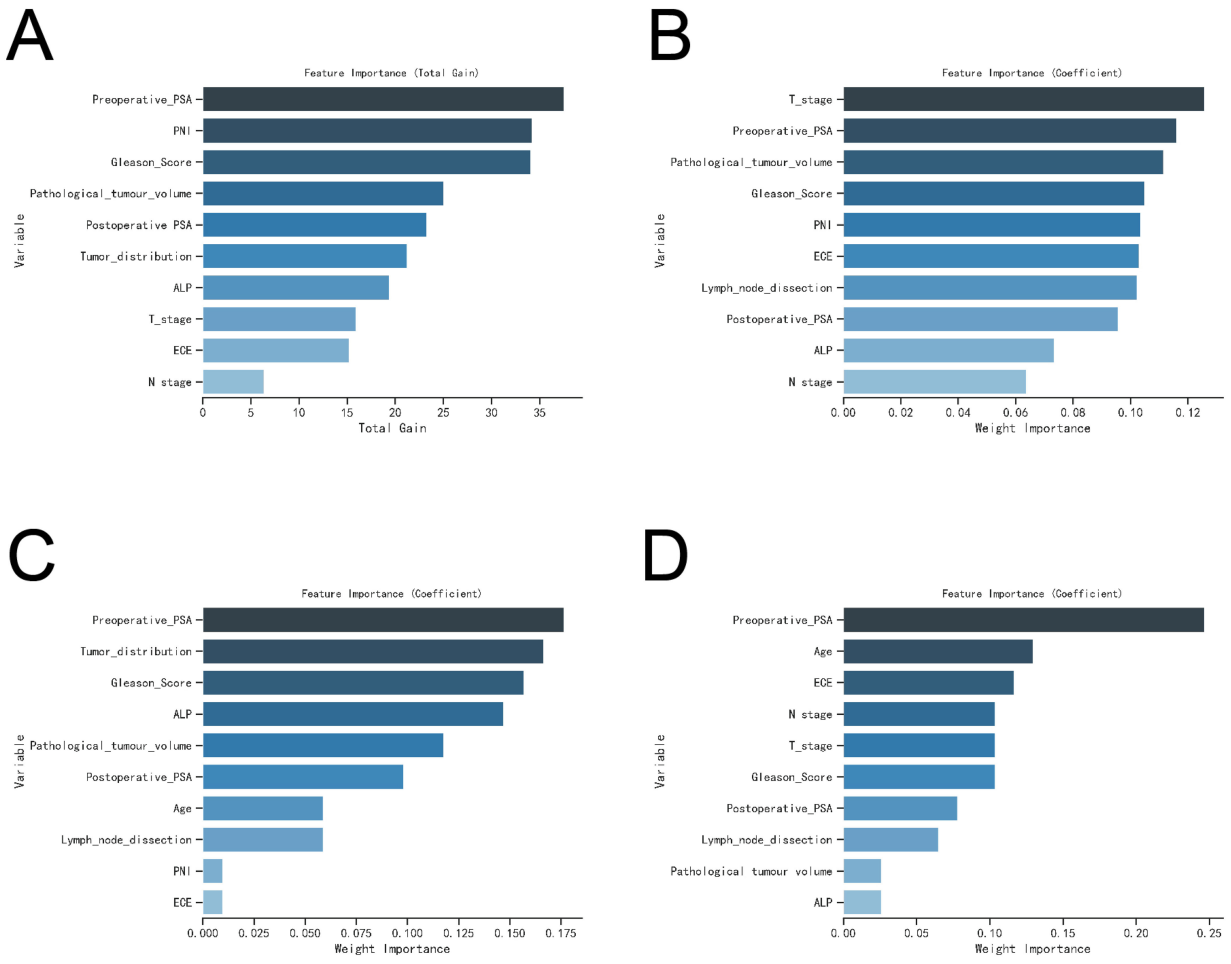
The variable influence factor ranking plots of the four models. **(A)** Variable importance ranking diagram of XGBoost model. **(B)** Variable importance ranking diagram of RF model. **(C)** Variable importance ranking diagram of SVM model. **(D)** Variable importance ranking diagram of KNN model.

To assess their impact on postoperative BM risk factors, we rigorously evaluated the performance of the XGBoost, Random Forest (RF), Support Vector Machine (SVM), and K-Nearest Neighbor (KNN) models. The analysis identified key factors contributing to postoperative BM risk, including ALP levels, Gleason Score, ECE invasion, pathological tumor volume, and both preoperative and postoperative PSA levels (see **Figures 5A–D**).

### Machine learning modeling and evaluation

**Figure 6:**
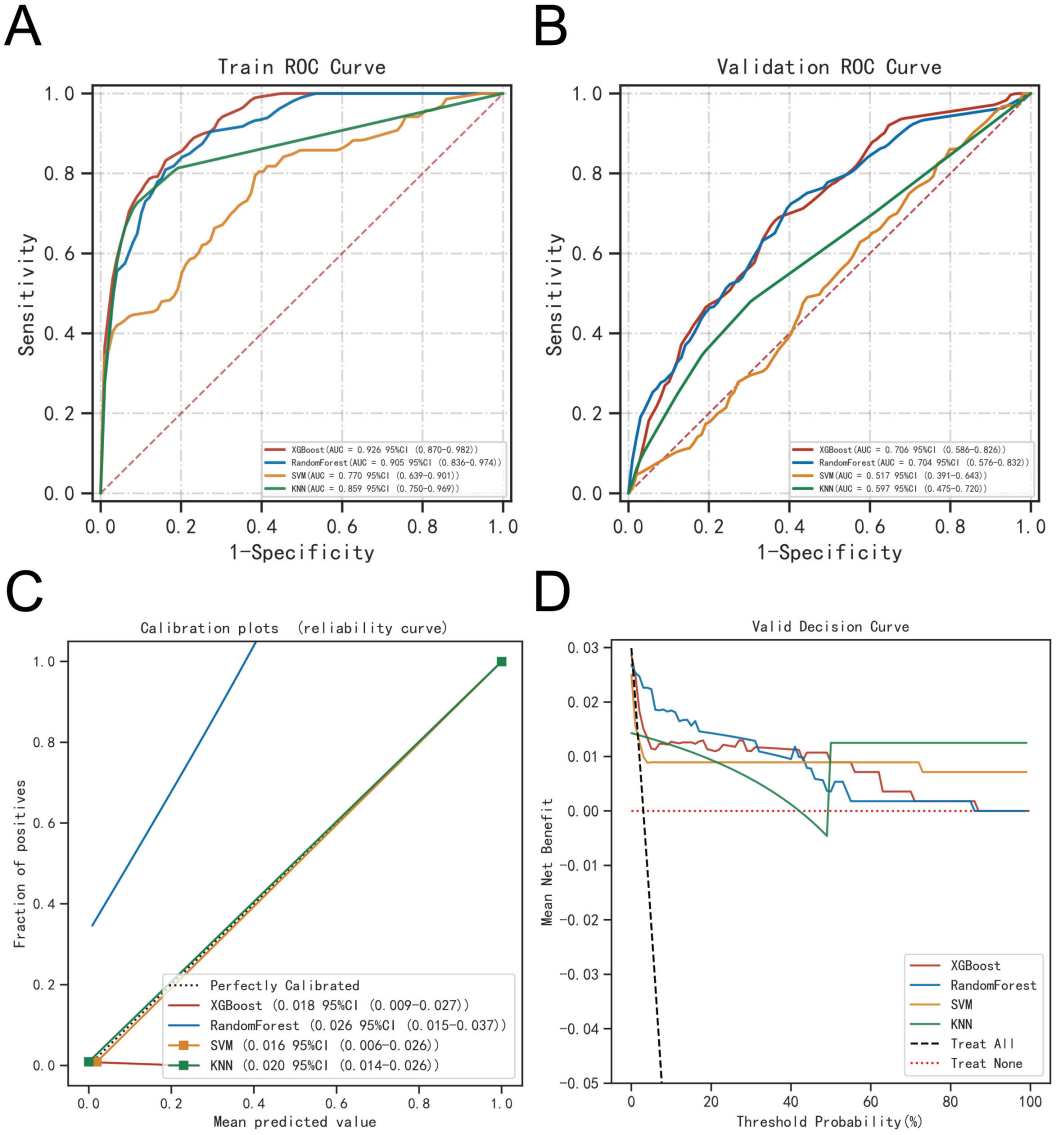
Evaluation of the four models for predicting BM. **(A)** ROC curves for the training set of the four models. **(B)** ROC curves for the validation set of the four models. **(C)** Calibration plots of the four models. The 45°dotted line on each graph represents the perfect match between the observed (y-axis) and predicted (x-axis) complication probabilitys. A closer distance between two curves indicates greater accuracy. **(D)** DCA curves of the four models. The four solid lines representing the four machine learning models have the intersection with the “all” curve as the starting point and the intersection with the “none” curve as the node within which the corresponding patient can benefit.

The ROC curve analysis demonstrated that the XGBoost model outperformed the other three models, achieving an AUC of 0.926 for the training set and 0.706 for the validation set (see **Table 2**; **Figures 6A, B**). Calibration curves for all models closely matched the ideal curves, indicating a strong alignment between predicted and actual outcomes (**Figure 6C**). Decision curve analysis (DCA) illustrated that each model provided a net clinical benefit compared to both the all-therapy and no-therapy scenarios (**Figure 6D**).

**Table 2:** Evaluation of the performance of the four models.

		AUC (95%CI)	Accuracy (95%CI)	Sensitivity (95%CI)	Specificity (95%CI)
KNN	training set	0.859 (0.750-0.969)	0.969 (0.964-0.974)	0.786(0.718-0.853)	0.916 (0.862-0.971)
	validation set	0.597 (0.475-0.720)	0.906 (0.892-0.920)	0.341(0.088-0.594)	0.862 (0.764-0.960)
XGBoost	training set	0.926 (0.870-0.982)	0.847 (0.773-0.921)	0.880(0.835-0.926)	0.829 (0.755-0.904)
	validation set	0.706 (0.586-0.826)	0.687 (0.661-0.713)	0.693(0.557-0.829)	0.664 (0.505-0.822)
RF	training set	0.905 (0.836-0.974)	0.833 (0.783-0.883)	0.854(0.810-0.897)	0.817 (0.767-0.867)
	validation set	0.704 (0.576-0.832)	0.723 (0.635-0.810)	0.676(0.589-0.763)	0.680 (0.594-0.765)
SVM	training set	0.770 (0.639-0.901)	0.731 (0.592-0.870)	0.787(0.577-0.996)	0.719 (0.564-0.875)
	validation set	0.517 (0.391-0.643)	0.783 (0.610-0.955)	0.845(0.686-1.004)	0.335 (0.134-0.536)

AUC, area under the curve; RF, random forest; XGBoost, extreme gradient boosting; SVM, support vector machine; KNN, k-nearest neighbor algorithm; CI, confidence interval.

**Figure 7:**
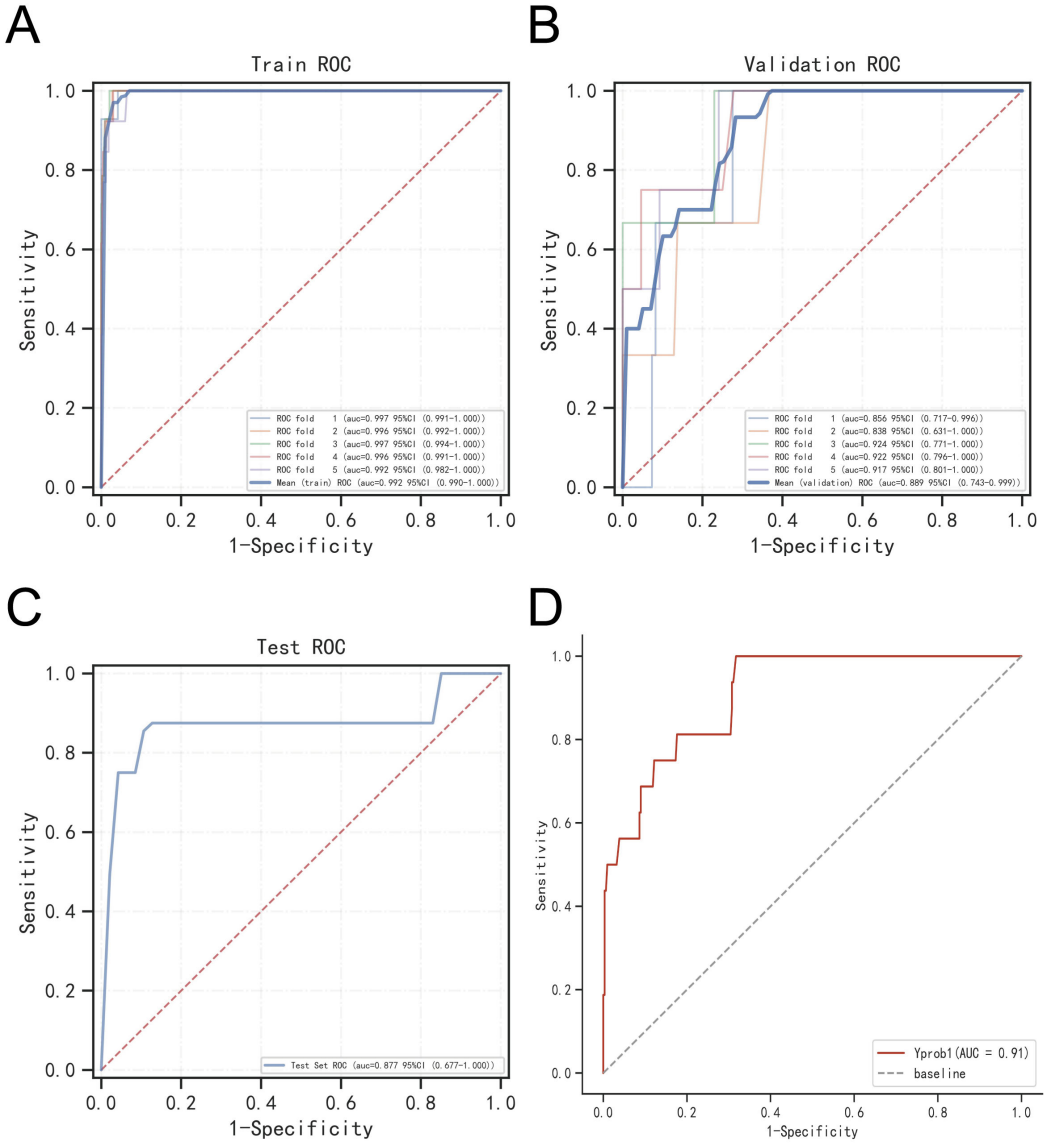
Validation of XGBoost model. **(A)** ROC curve of XGBoost model for the training set. **(B)** ROC curve of XGBoost model for the validation set. **(C)** ROC curve of XGBoost model for the test set. **(D)** External validation of XGBoost model.

To assess model generalizability, we employed k-fold cross-validation. For the XGBoost model, with a test set consisting of 240 cases (30.00%), and the remaining samples used for 5-fold cross-validation, the model achieved an AUC of 0.8914 ± 0.0367 for the validation set and an AUC of 0.8772 for the test set, with an accuracy of 0.9417 (**Figures 7A–C**). The Random Forest (RF) model produced an AUC of 0.7830 ± 0.0923 for the validation set and an AUC of 0.8610 for the test set, with an accuracy of 0.9917. The Support Vector Machine (SVM) model recorded an AUC of 0.8376 ± 0.1205 for the validation set and an AUC of 0.8739 for the test set, with an accuracy of 0.8292. Lastly, the K-Nearest Neighbor (KNN) model achieved an AUC of 0.7452 ± 0.1492 for the validation set and an AUC of 0.8723 for the test set, with an accuracy of 0.9917. Based on these comprehensive evaluations, the XGBoost algorithm was identified as the most effective model for this study.

### External validation of models

The ROC curve analysis for the external validation set revealed an AUC of 0.91, highlighting the model’s excellent accuracy in identifying the disease (**Figure 7D**).

### Model interpretation

**Figure 8:**
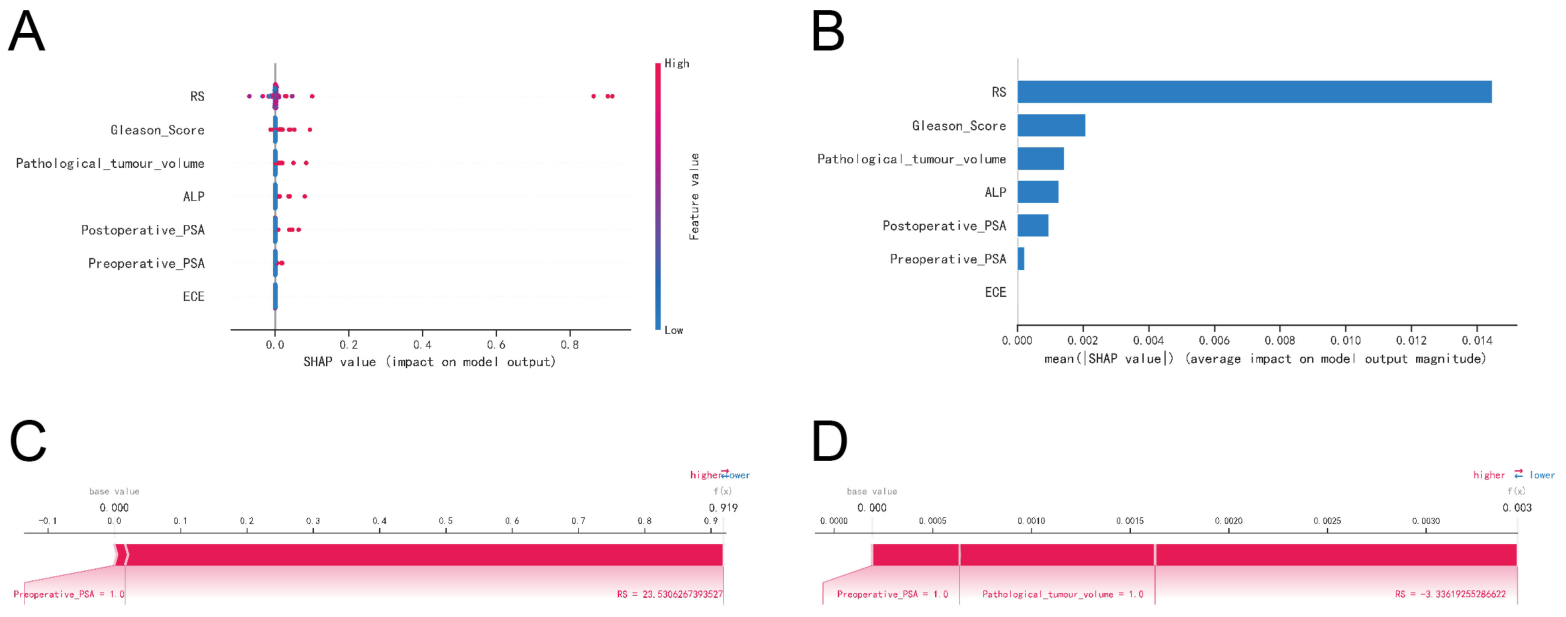
Interpretability of machine learning models. **(A)** SHAP summary plot. Risk factors are arranged along the y-axis based on their importance, which is given by the mean of their absolute Shapley values. The higher the risk factor is positioned in the plot, the more important it is for the model. **(B)** Characteristic importance ranking plot. **(C)** Patient I’s SHAP force plot. The contributing variables are arranged in the horizontal line, sorted by the absolute value of their impact. Blue represents features that have a negative effect on disease prediction, with a decrease in SHAP values; red represents features that have a positive effect on disease prediction, with an increase in SHAP values. **(D)** Patient II’s SHAP force plot.

The SHAP summary plot highlighted the following risk factors for bone metastasis (BM) in prostate cancer, ordered by their importance: Radscore (higher), Gleason Score (higher), pathological tumor volume (larger), elevated ALP levels, elevated postoperative PSA, elevated preoperative PSA, and extracapsular extension (ECE) invasion of the tumor (**Figures 8A, B**).

The SHAP force plots offered detailed insights into the model’s predictions for two patients with prostate cancer BM. For Patient 1, the model predicted a 0.919 probability of BM, driven primarily by a preoperative PSA ≥ 20 ng/ml and a Radscore ≥ 23.5306 (**Figure 8C**). For Patient 2, the model estimated a much lower probability of 0.003 for BM, influenced by a preoperative PSA ≥ 0.2 ng/ml, a pathological tumor volume ≥ 60 mm², and a Radscore ≥ -3.3362 (**Figure 8D**).

## Discussion

This study assessed risk prediction models for prostate cancer bone metastasis (BM) using four distinct machine learning algorithms. Among these, the XGBoost algorithm demonstrated superior performance, characterized by its versatility and interpretability (10). XGBoost outperformed the Random Forest (RF) algorithm in processing efficiency, thanks to its advanced features like multithreaded processing and feature-based splitting, which enhance training and prediction speeds (11). Unlike Support Vector Machine (SVM) and K-Nearest Neighbors (KNN), XGBoost supports both L1 and L2 regularization, which effectively controls model complexity and reduces overfitting risks (10, 12). Consequently, XGBoost was chosen for constructing the predictive model for postoperative BM in prostate cancer patients.

According to the “seed-soil” hypothesis by Paget, tumor metastasis involves the implantation of tumor cells in a favorable microenvironment, facilitating their growth and spread (13). This process is complex and involves the proliferation of neoplastic cells, their detachment from the primary tumor, migration, and secondary growth (14, 15). The predictive model developed in this study, leveraging imaging data, offers high precision in forecasting BM onset, which is crucial for early detection of high-risk patients.

Our analysis included 109 imaging histological attributes to predict BM occurrence in prostate cancer using nuclear magnetic resonance imaging data. After rigorous selection, six key attributes were chosen: original_glszm_LargeAreaHighGrayLevelEmphasis, original_glrlm_ShortRunHighGrayLevelEmphasis, original_gldm_SmallDependenceHighGrayLevelEmphasis, original_glcm_SumAverage, original_glcm_DifferenceEntropy, and original_shape_Maximum2DDiameterColumn. This approach integrates both clinical and imaging data, offering a more comprehensive risk assessment compared to studies focused solely on clinical parameters. This integration is crucial for early intervention and tailored therapeutic strategies.

SHAP analysis further elucidated the risk factors associated with BM in prostate cancer, highlighting the significant roles of pathological tumor volume, ECE invasion, ALP levels, Gleason score, and PSA values (preoperative and postoperative). The link between pathological tumor volume and malignancy is well-documented, with larger tumors often exhibiting increased invasiveness and a higher propensity for metastasis (16). Larger tumors require greater blood supply, leading to tumor angiogenesis and increased potential for metastatic spread (17, 18). Additionally, the Gleason score provides a detailed assessment of tumor aggressiveness, which is crucial for determining appropriate treatment (19). Our findings align with previous research, reinforcing the significance of these factors in predicting BM risk.

The study also noted that prostate cancer cells with ECE invasion are more likely to metastasize, potentially due to increased surgical challenges and inflammatory responses during surgery (20). This observation is consistent with Guerra et al.’s research, which found that ECE invasion enhances tumor cell motility, facilitating metastasis (21).

Elevated ALP levels were identified as a significant risk factor for BM. ALP, an enzyme involved in various biological processes, including bone metabolism, is released in response to tumor-induced bone resorption (22). Elevated ALP levels have been linked to poorer survival outcomes in cancer patients (23).

Our study utilized two distinct sample cohorts to validate the predictive model for BM in prostate cancer, emphasizing the role of PSA levels (both preoperative and postoperative) as crucial risk factors. Elevated PSA levels correlate with increased tumor activity and metastatic potential, impacting patient prognosis (24). Our prostate cancer bone metastasis (PCBM) risk prediction model has wide applications across various clinical settings, including preoperative planning, intraoperative guidance, and postoperative management. During initial physical exams or outpatient screenings, the model enables early identification of high-risk individuals and recommends further imaging studies, such as bone scans or CT scans. At the preoperative stage, the model assists in assessing the risk of prostate cancer bone metastasis when preparing for related treatments. For high-risk patients, the model helps formulate detailed treatment strategies, including preoperative adjustments to anticoagulation or metabolic therapy. For asymptomatic patients diagnosed with prostate cancer bone metastasis, the model supports the assessment of recurrence or complication risks, helping clinicians determine whether preventive interventions are necessary. Additionally, for high-risk prostate cancer bone metastasis patients, the model guides optimization of preoperative management, such as implementing enhanced immunotherapy strategies. During treatment, the model provides critical insights, aiding physicians in designing treatment plans or increasing treatment intensity, such as adjusting radiation therapy or chemotherapy protocols, thereby reducing the risks associated with bone metastasis. Postoperatively, the model facilitates personalized surveillance plans, including routine imaging and biochemical monitoring, particularly suitable for long-term follow-up of prostate cancer bone metastasis patients to assess the risk of recurrence or other bone-related complications.

While parametric regression models have been effective for predicting metastasis in prostate cancer, they may not adequately address the complexity of clinical data with nonlinear relationships (25, 26). This study’s use of the XGBoost algorithm provided a robust predictive model for BM post-radical prostatectomy, demonstrating high performance and clinical utility. However, limitations include the exclusion of imaging data from other modalities and the lack of consideration for targeted therapies (27–31). Future research should incorporate multicenter studies to enhance the robustness and reliability of these findings. It is well known that many aspects of postoperative management, such as medication, diet, and daily activities, can influence the occurrence of prostate cancer bone metastasis. This study primarily focused on exploring the potential of preoperative MRI imaging data in predicting prostate cancer bone metastasis, while overlooking the impact of other factors on tumor metastasis. In future research, we plan to develop a comprehensive prediction model that incorporates postoperative management and other clinical factors to further enhance the model’s predictive ability and clinical applicability.

## Conclusion

In this study, we developed a predictive model integrating imaging histology and machine learning algorithms to estimate the risk of tumor bone metastasis (BM) following radical prostatectomy for prostate cancer. The model demonstrated high predictive accuracy and substantial clinical utility, providing surgeons with a valuable tool for early diagnosis and intervention. Key findings indicate that BM remains a significant challenge for prostate cancer patients, with its risk strongly correlated with elevated alkaline phosphatase (ALP) levels, larger tumor sizes, higher Gleason scores, extraprostatic extension (ECE) invasion, and increased prostate-specific antigen (PSA) levels both preoperatively and postoperatively.

## Data Availability

The original contributions presented in the study are included in the article/**Supplementary Material**. Further inquiries can be directed to the corresponding author.

## References

[1] Plata Bello A Concepcion Masip T. Prostate cancer epidemiology. Arch Esp Urol. (2014) 67:373–82. 24914835

[2] Conti DV Darst BF Moss LC Saunders EJ Sheng X Chou A. Trans-ancestry genome-wide association meta-analysis of prostate cancer identifies new susceptibility loci and informs genetic risk prediction. Nat Genet. (2021) 53:65–75. doi: 10.1038/s41588-020-00748-0 33398198 PMC8148035

[3] Yamamichi G Kato T Uemura M Nonomura N. Diagnosing and prognosing bone metastasis in prostate cancer: clinical utility of blood biomarkers. Anticancer Res. (2023) 43:283–90. doi: 10.21873/anticanres.16161 36585196

[4] Nakanishi K Tanaka J Nakaya Y Maeda N Sakamoto A Nakayama A. Whole-body mri: detecting bone metastases from prostate cancer. Jpn J Radiol. (2022) 40:229–44. doi: 10.1007/s11604-021-01205-6 PMC8891104 34693502

[5] Coleman RE. Clinical features of metastatic bone disease and risk of skeletal morbidity. Clin Cancer Res. (2006) 12:6243s–9s. doi: 10.1158/1078-0432.Ccr-06-0931 17062708

[6] Raboy A Ferzli G Albert P. Initial experience with extraperitoneal endoscopic radical retropubic prostatectomy. Urology. (1997) 50:849–53. doi: 10.1016/s0090-4295(97)00485-8 9426712

[7] Hofman MS Lawrentschuk N Francis RJ Tang C Vela I Thomas P. Prostate-specific membrane antigen pet-ct in patients with high-risk prostate cancer before curative-intent surgery or radiotherapy (Propsma): A prospective, randomised, multicentre study. Lancet. (2020) 395:1208–16. doi: 10.1016/s0140-6736(20)30314-7 32209449

[8] Dong J Feng T Thapa-Chhetry B Cho BG Shum T Inwald DP. Machine learning model for early prediction of acute kidney injury (Aki) in pediatric critical care. Crit Care. (2021) 25:288. doi: 10.1186/s13054-021-03724-0 34376222 PMC8353807

[9] Wu CT Li GH Huang CT Cheng YC Chen CH Chien JY. Acute exacerbation of a chronic obstructive pulmonary disease prediction system using wearable device data, machine learning, and deep learning: development and cohort study. JMIR Mhealth Uhealth. (2021) 9:e22591. doi: 10.2196/22591 33955840 PMC8138712

[10] Tseng PY Chen YT Wang CH Chiu KM Peng YS Hsu SP. Prediction of the development of acute kidney injury following cardiac surgery by machine learning. Crit Care. (2020) 24:478. doi: 10.1186/s13054-020-03179-9 32736589 PMC7395374

[11] Liu W Wang S Ye Z Xu P Xia X Guo M. Prediction of lung metastases in thyroid cancer using machine learning based on seer database. Cancer Med. (2022) 11:2503–15. doi: 10.1002/cam4.4617 PMC9189456 35191613

[12] Yue S Li S Huang X Liu J Hou X Zhao Y. Machine learning for the prediction of acute kidney injury in patients with sepsis. J Transl Med. (2022) 20:215. doi: 10.1186/s12967-022-03364-0 35562803 PMC9101823

[13] Mendoza M Khanna C. Revisiting the seed and soil in cancer metastasis. Int J Biochem Cell Biol. (2009) 41:1452–62. doi: 10.1016/j.biocel.2009.01.015 PMC7251640 19401145

[14] Liang Y Zhang H Song X Yang Q. Metastatic heterogeneity of breast cancer: molecular mechanism and potential therapeutic targets. Semin Cancer Biol. (2020) 60:14–27. doi: 10.1016/j.semcancer.2019.08.012 31421262

[15] Ni J Wang X Stojanovic A Zhang Q Wincher M Bühler L. Single-cell rna sequencing of tumor-infiltrating nk cells reveals that inhibition of transcription factor hif-1α Unleashes nk cell activity. Immunity. (2020) 52:1075–87.e8. doi: 10.1016/j.immuni.2020.05.001 32445619

[16] Liao CP Booker RC Brosseau JP Chen Z Mo J Tchegnon E. Contributions of inflammation and tumor microenvironment to neurofibroma tumorigenesis. J Clin Invest. (2018) 128:2848–61. doi: 10.1172/jci99424 PMC6025974 29596064

[17] Kurebayashi Y Matsuda K Ueno A Tsujikawa H Yamazaki K Masugi Y. Immunovascular classification of hcc reflects reciprocal interaction between immune and angiogenic tumor microenvironments. Hepatology. (2022) 75:1139–53. doi: 10.1002/hep.32201 34657298

[18] Wolf JE Jr. Hubler WR Jr. Tumor angiogenic factor and human skin tumors. Arch Dermatol. (1975) 111:321–7. doi: 10.1001/archderm.1975.01630150041003 1091213

[19] Sehn JK. Prostate cancer pathology: recent updates and controversies. Mo Med. (2018) 115:151–5. PMC6139855 30228708

[20] Kaiser A Haskins C Siddiqui MM Hussain A D’Adamo C. The evolving role of diet in prostate cancer risk and progression. Curr Opin Oncol. (2019) 31:222–9. doi: 10.1097/cco.0000000000000519 PMC7379157 30893147

[21] Guerra A Flor-de-Lima B Freire G Lopes A Cassis J. Radiologic-pathologic correlation of prostatic cancer extracapsular extension (Ece). Insights Imaging. (2023) 14:88. doi: 10.1186/s13244-023-01428-3 37191739 PMC10188796

[22] Guo Y Xie YQ Gao M Zhao Y Franco F Wenes M. Metabolic reprogramming of terminally exhausted cd8(+) T cells by il-10 enhances anti-tumor immunity. Nat Immunol. (2021) 22:746–56. doi: 10.1038/s41590-021-00940-2 PMC7610876 34031618

[23] Hung HY Chen JS Chien Y Tang R Hsieh PS Wen S. Preoperative alkaline phosphatase elevation was associated with poor survival in colorectal cancer patients. Int J Colorectal Dis. (2017) 32:1775–8. doi: 10.1007/s00384-017-2907-4 29030683

[24] Hamdy FC Donovan JL Lane JA Metcalfe C Davis M Turner EL. Fifteen-year outcomes after monitoring, surgery, or radiotherapy for prostate cancer. N Engl J Med. (2023) 388:1547–58. doi: 10.1056/NEJMoa2214122 36912538

[25] Rocco B Sighinolfi MC Sandri M Puliatti S Bianchi G. A novel nomogram for predicting ece of prostate cancer. BJU Int. (2018) 122:916–8. doi: 10.1111/bju.14503 30460784

[26] Gandaglia G Ploussard G Valerio M Mattei A Fiori C Fossati N. A novel nomogram to identify candidates for extended pelvic lymph node dissection among patients with clinically localized prostate cancer diagnosed with magnetic resonance imaging-targeted and systematic biopsies. Eur Urol. (2019) 75:506–14. doi: 10.1016/j.eururo.2018.10.012 30342844

[27] Chaudhuri D Sasaki K Karkar A Sharif S Lewis K Mammen MJ. Corticosteroids in covid-19 and non-covid-19 ards: A systematic review and meta-analysis. Intensive Care Med. (2021) 47:521–37. doi: 10.1007/s00134-021-06394-2 PMC8054852 33876268

[28] Raison N Servian P Patel A Santhirasekaram A Smith A Yeung M. Is tumour volume an independent predictor of outcome after radical prostatectomy for high-risk prostate cancer? Prostate Cancer Prostatic Dis. (2023) 26:282–6. doi: 10.1038/s41391-021-00468-4 PMC10247356 34845306

[29] Zheng D Chen H Davids J Bryant M Lucas A. Serpins for diagnosis and therapy in cancer. Cardiovasc Hematol Disord Drug Targets. (2013) 13:123–32. doi: 10.2174/1871529x11313020005 23988000

[30] Guerra A Alves FC Maes K Joniau S Cassis J Maio R. Early biomarkers of extracapsular extension of prostate cancer using mri-derived semantic features. Cancer Imaging. (2022) 22:74. doi: 10.1186/s40644-022-00509-8 36550525 PMC9784252

[31] Schoppet M Shanahan CM. Role for alkaline phosphatase as an inducer of vascular calcification in renal failure? Kidney Int. (2008) 73:989–91. doi: 10.1038/ki.2008.104 18414436

